# miRNA‐449c‐5p regulates the JAK‐STAT pathway in inhibiting cell proliferation and invasion in human breast cancer cells by targeting ERBB2

**DOI:** 10.1002/cnr2.1974

**Published:** 2024-02-13

**Authors:** Li Li, Yangqiurong Zhang, Kunxian Yang, Wei Liu, Ziting Zhou, Ying Xu

**Affiliations:** ^1^ Department of Breast and Thyroid Surgery The First People's Hospital of Yunnan Province, The Affiliated Hospital of Medical College, Kunming University of Science and Technology Kunming China

**Keywords:** breast cancer, ERBB2, JAK/STAT signaling, miR‐449c‐5p

## Abstract

**Background:**

Breast cancer is a highly prevalent disease worldwide, and early diagnosis and treatment could reduce the mortality rate of breast cancer patients. microRNAs (miRNA) have been shown to regulate the occurrences and progression of many types of cancers. Thus, it is crucial to find novel biomarkers in breast cancer. miR‐449c‐5p acted as a biomarker in non‐small cell lung cancer, gastric carcinoma, and so forth. ERBB2 is an ideal target for breast cancer therapy. However, the molecular mechanisms between miR‐449c‐5p and ERBB2 in breast cancer remain poorly understood. Our study focused on the regulatory role of miR‐449c‐5p in breast cancer and its targeting relationship with ERBB2.

**Methods:**

The miR‐449c‐5p expression in breast cancer tissue and normal tissue was searched from the online database (Starbase). The clinical prognosis of miR‐449c‐5p and ERBB2 was predicted by using the Kaplan–Meier analysis method. The expression of miR‐449c‐5p mimics and inhibitors was measured by qRT‐PCR. T47D cells were transfected with miR‐449c‐5p mimics and miR‐449c‐5p inhibitors. After that, CCK‐8, colony formation assays and Transwell assays were used to evaluate the cell proliferation ability, migration and invasion. Whether ERBB2 was the target gene of the miR‐449c‐5p was predicted by Starbase and verified by dual‐luciferase activity assay. In addition, protein levels and the relationship between signalling pathways were measured and validated using western blotting analysis.

**Results:**

We confirmed that miR‐449c‐5p was highly expressed in breast cancer tissue, and its downregulation was linked with poor prognosis. Overexpression of miR‐449c‐5p inhibited the proliferation, migration and invasion of breast cancer cells. ERBB2 was a target of miR‐449c‐5p. The invasion, migration, and proliferation of breast cancer cells were inhibited by miR‐449c‐5p/ERBB2 through JAK‐STAT.

**Conclusion:**

This study demonstrated that miR‐449c‐5p inhibits breast cancer cell proliferation, migration and invasion by targeting ERBB2 via JAK/STAT, which means miR‐449c‐5p, is a potential biomarker for breast cancer and provides a novel insight for diagnosis.

## 1 INTRODUCTION

Breast cancer is a prevalent malignancy worldwide. It brings serious threats to women's health. Since 2019, breast cancer has emerged as the most frequently diagnosed cancer in the world.^1^ According to global cancer statistics,^2^ almost 10.0 million cancer‐related fatalities occurred in 2020, and female breast cancer accounted for 6.9%. Effective treatment options encompass surgical intervention, chemotherapy, and radiation therapy.^3^ These therapeutic interventions increase the survival rate of breast cancer patients. Consistently, research reported that mortality could be decreased by early diagnosis and therapy.^4^ The reviews indicated that the rate of recovery for breast cancer is approximately 70%–80% during the initial stages and non‐metastatic phase.^5^

Conventionally, breast cancer is diagnosed and predicted using various biological characteristics, for example, histological grade, lymph node status, hormone receptor status, and human epidermal growth factor receptor type 2 (HER2) status.^6^ However, there have been cases where patients have exhibited varying clinical outcomes, primarily due to the tumor relapse and distant metastases, which remain the primary factors leading to mortality in breast cancer.^7^ Therefore, it is important to discover more biomarkers for early diagnosis. Furthermore, there is a strong desire to identify the progression mechanisms of breast cancer and explore novel therapeutic targets.

miRNAs are non‐coding RNAs that participate in almost all cellular processes; they have ability to impact the main gene expression in malignancy. They also bind to specific mRNA to regulate post‐transcriptional gene expression.^8^ The irregular expression of miRNAs may increase the cell proliferation, angiogenesis, migration and apoptosis.^9^ The onset and progression of cancer are correlated with aberrant miRNA expression. Multiple miRNA species have been used to diagnose cancers such as lung, colorectal, prostate, and breast cancer.^10^

The recent research demonstrated that miR‐449 was a potential regulator in several types of cancer.^11^ The specific role and molecular targets of miR‐449c‐5p have been extensively studied in several types of cancer, including non‐small cell lung cancer and gastric carcinoma.^12^ Research in non‐cancer disease has demonstrated that miR‐449c‐5p suppresses the process of bone formation in valve interstitial cells, making it a potential target for calcific aortic valve disease (CAVD).^13^ Moreover, the study verified that miR‐449c‐5p is directly targeted on SOX4 in hepatocellular carcinoma.^14^ However, there has been limited research on the clinical role, specific targets, and regulatory pathways of miR‐449c‐5p in breast cancer.

In this study, we found the expression level of miR‐449c‐5p in breast cancer tissue is up‐regulated compared with normal tissue from publicly available sources in the Starbase database. We also identified the influences on the proliferation of breast cancer cells due to miR‐449c‐5p overexpression. Then, we predicted the target relationship between miR‐449c‐5p and ERBB2. However, it remains largely unknown how miR‐449c‐5p affects breast cancers. Herein, we demonstrated that miR‐449‐5p decreases the expression level of ERBB2, hence inhibiting cell proliferation and invasion through the JAK/STAT pathway in breast cancer. Our results might provide potential therapeutic and diagnostic targets for breast cancer.

## 2 MATERIALS AND METHODS

### 2.1 Differential expression and survival analysis

A total of 1085 breast cancer and 104 normal tissues were used to predict the expression level of miR‐449c‐5p, provided by Starbase (ENCORI). Also, the overall survival for miR‐449c‐5p in breast cancer was detected by Starbase through 795 low‐expression samples and 287 high‐expression samples. Moreover, Starbase was employed to examine the differential expression level in breast cancer using 1104 malignant and 113 normal samples. (https://rnasysu.com/encori/index.php).

To comprehensively assess the effects of ERBB2, distant metastasis, disease‐free survival, relapse‐free survival, disease‐specific survival and overall survival were searched from PrognoScan. (http://dna00.bio.kyutech.ac.jp/PrognoScan/index.html).

### 2.2 Cell culture and transfection

T47D cells were purchased from the American Type Culture Collection (ATCC) (Manassas, VA). RPMI 1640 or DMEM media was cultured, supplemented with 10% fetal bovine serum at 37 °C in a humidified atmosphere with 5% CO_2_.

The miR‐449c‐5p mimics, inhibitors, and corresponding control miRNAs (NC‐miRNA) were designed and synthesized by RiboBio (Guangzhou, China), the sequence information was shown in Table 1. T47D cells were seeded into 6‐well plates with 5 × 10^4^ cells each well and cultured to 60%–70% confluence for transfection. Further, cells were transfected using Lipofectamine 2000 (Invitrogen) according to the manufacturer's instructions. After 48 h, the RNA levels were detected and quantified by qRT‐PCR.

**TABLE 1:** Sequence information.

Name	Sequence
miR‐449c‐5p mimics	Forward: UAGGCAGUGUAUUGCUAGCGGCUGU Reverse: ACAGCCGCUAGCAAUACACUGCCUA
miR‐449c‐5p inhibitor	Forward: ACAGCCGCUAGCAAUACACUGCCUA 2'Ome
NC‐miRNA	Forward: UCACAACCUCCUAGAAAGAGUAGA Reverse: UCUACUCUUUCUAGGAGGUUGUGA

pcDNA3.1 vector was inserted into construct ERBB2 overexpressing plasmid. Synthetic vectors were transfected into T47D cells by using Lipofectamine 3000 (Invitrogen, Carlsbad, CA) according to the manufacturer's manual.

### 2.3 qPCR

TRIzol reagent was used to extract total RNA (Invitrogen, Thermo Fisher Scientific). Following real‐time PCR experiments, the SYBR Green PCR Kit (Thermo Fisher Scientific) was used. The following are the thermal cycling conditions: 95 °C for 2 min, then 44 cycles of denaturation at 95 °C for 10 s and annealing/extension at 56 °C for 1 min. The relative expression level of miR‐449c‐5p was compared to U6 expression and was calculated using the 2^−ΔΔCt^ method. The primer sequences are miR‐449c‐5p forward 5'‐CAGTGTATTGCTAGCGGCTGT‐3′. U6 forward 5'‐GCTTCGGCAGCACATATACTAA‐3′.

### 2.4 CCK‐8 assay and colony formation

The CCK‐8 assay kit (CCK‐8; Dojindo, Kumamoto, Japan) was used to evaluate breast cancer cell viability. Cells were transfected in 96‐well plates, cultured in suitable conditions for 48 h, and then treated with 10 μL CCK‐8 solution in each well. After incubation at 37 °C for 2 h, the measured absorbance was at 450 nm. The transfected T47D cells were seeded in a 12‐well culture plate for colony formation assays and incubated at 37 °C for a week. Then, it was fixed with methanol and stained with 0.1% crystal violet. The number of colonies was counted with ImageJ.

### 2.5 Cell migration and invasion

T47D cells were planted in 6‐well plates for the wound healing experiment. A sterile pipette tip was used to scrape the cells and obtained images at 0 h and 48 h by microscope. Wound width was measured and analysed by ImageJ. For Transwell assays, 24‐well 8 μm pore size Transwell chambers were used, then seeded T47D cells into the upper chamber with Matrigel (Corning, Tewksbury, MA). The lower chamber was contained with 10% FBS, and 200 μL cell suspension was added into the upper chamber; after 24 h, the cells in the upper chamber were gently wiped with a cotton swab. T47D cells were fixed by adding 4% paraformaldehyde (Biosharp, China) to 6‐well plates and then stained. Finally, ImageJ was used to examine the average number of migrating cells based on microscope fields.

### 2.6 Targeted gene prediction and verification

The target genes of miR‐449c‐5p were predicted by a Starbase database (https://rnasysu.com/encori/index.php). Then, the dual‐luciferase reporter assay was to validate the relationship between ERBB2 and miR‐449c‐5p. The wild‐type luciferase reporter vector WT‐ERBB2 containing a binding site for miR‐449c‐5p was constructed into the PGL‐base vector, and the corresponding MUT vector was constructed. Cells were co‐transfected with the wild‐type and mutant ERBB2 3′‐UTR and miRNA mimics using Lipofectamine 2000 (Invitrogen). After 48 h, luciferase activity was measured using the Dual‐Luciferase Reporter Assay Kit (Promega, Madison, WI) according to the manufacturer's instructions. The activity of firefly luciferase was compared to that of Renilla luciferase.

### 2.7 Western blotting

The RIPA extraction reagent (Beyotime, Shanghai, China) were used to extract proteins. The protein was then separated from the sample buffer using SDS‐PAGE, transferred to PVDF membranes, and blocked for 1 h with 5% skim milk. Primary antibodies were provided from Abcam company, anti‐ERBB2 (1:1000, ab134182), anti‐JAK1 (1:1000, ab138005), anti‐JAK2 (1:1000, ab267373), anti‐STAT3 (1:1000, ab68153), anti‐STAT5 (1:1000, ab32364), anti‐GAPDH (1:1000, ab8245). Then, bands were visualized by a chemiluminescence (ECL) kit (Boster, China) and analysed by ImageJ.

### 2.8 Statistical analysis

The data is presented as mean ± SD. Student's *t*‐test, one‐way or two‐way analysis of variance was used to determine the difference between groups (ANOVA). GraphPad Prism 8.0 software (GraphPad Software, San Diego, CA) was used to generate the analysis results. The Kaplan–Meier method was used to generate survival curves, and the differences were assessed using a log‐rank test. *P* < .05 was considered statistical significance, and *P* < .01 and *P* < .001 were considered to have a high degree of statistical significance.

## 3 RESULTS

### 3.1 miR‐449c‐5p was upregulated in breast cancer tissues compared to normal tissues

**FIGURE 1:**
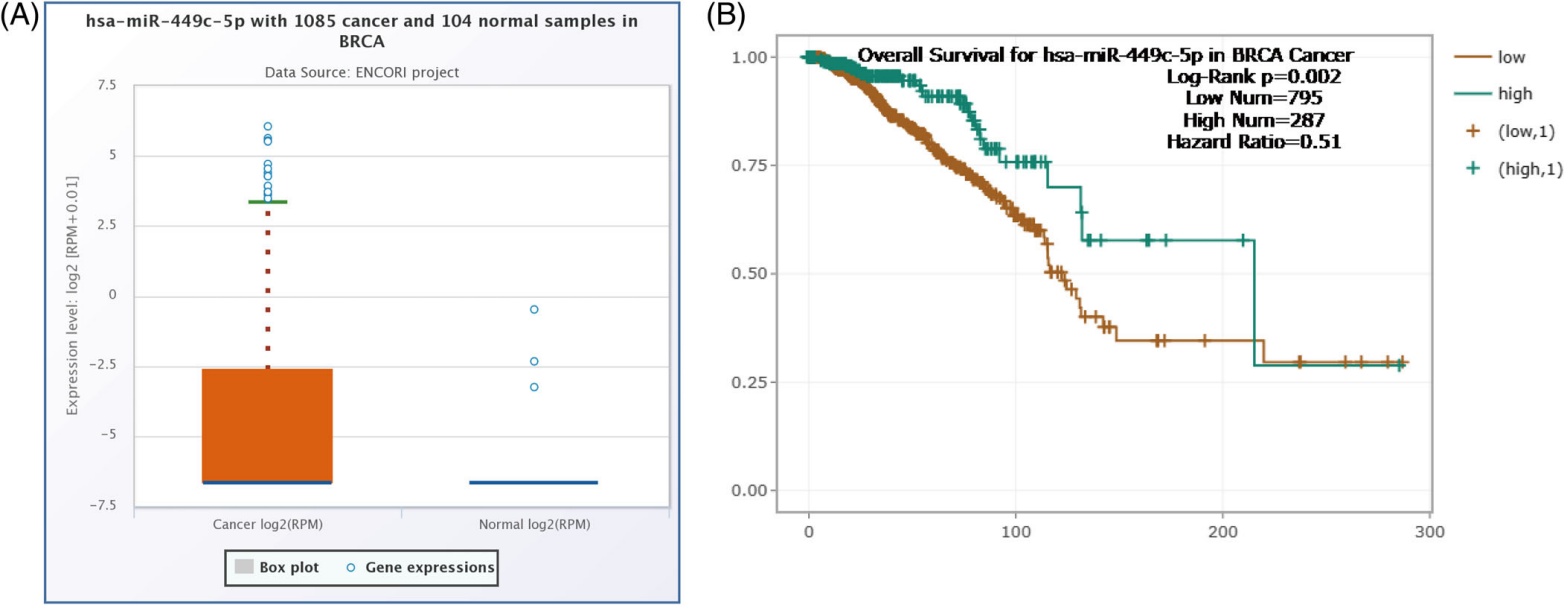
The expression level and the overall survival rate of miR‐449‐5p in breast cancer tissues. (A) miR‐449p‐5c expression in the breast cancer tissue compared with normal tissue. (B) Kaplan–Meier survival curves of breast cancer patients with low and high miR‐449c‐5p expression. Log‐Rank *p* = .02.

To investigate whether miR‐449c‐5p is correlated with the malignant behavior of breast cancer, we used Starbase to predict miR‐449c‐5p expression levels in breast cancer tissues and normal tissues. miR‐449‐5p was found to be significantly up‐regulated in breast cancer tissues (Figure 1A). Then, the Kaplan–Meier analysis and log‐rank analysis were used to evaluate the overall survival rate. The plot showed lower miR‐449c‐5p expression had a worse prognosis than those with higher expression (Figure 1B). These results suggest that miR‐449c‐5p might be a tumor inhibitor in breast cancer.

### 3.2 Overexpression of miR‐449c‐5p inhibited the proliferation, migration and invasion of breast cancer cells

**FIGURE 2:**
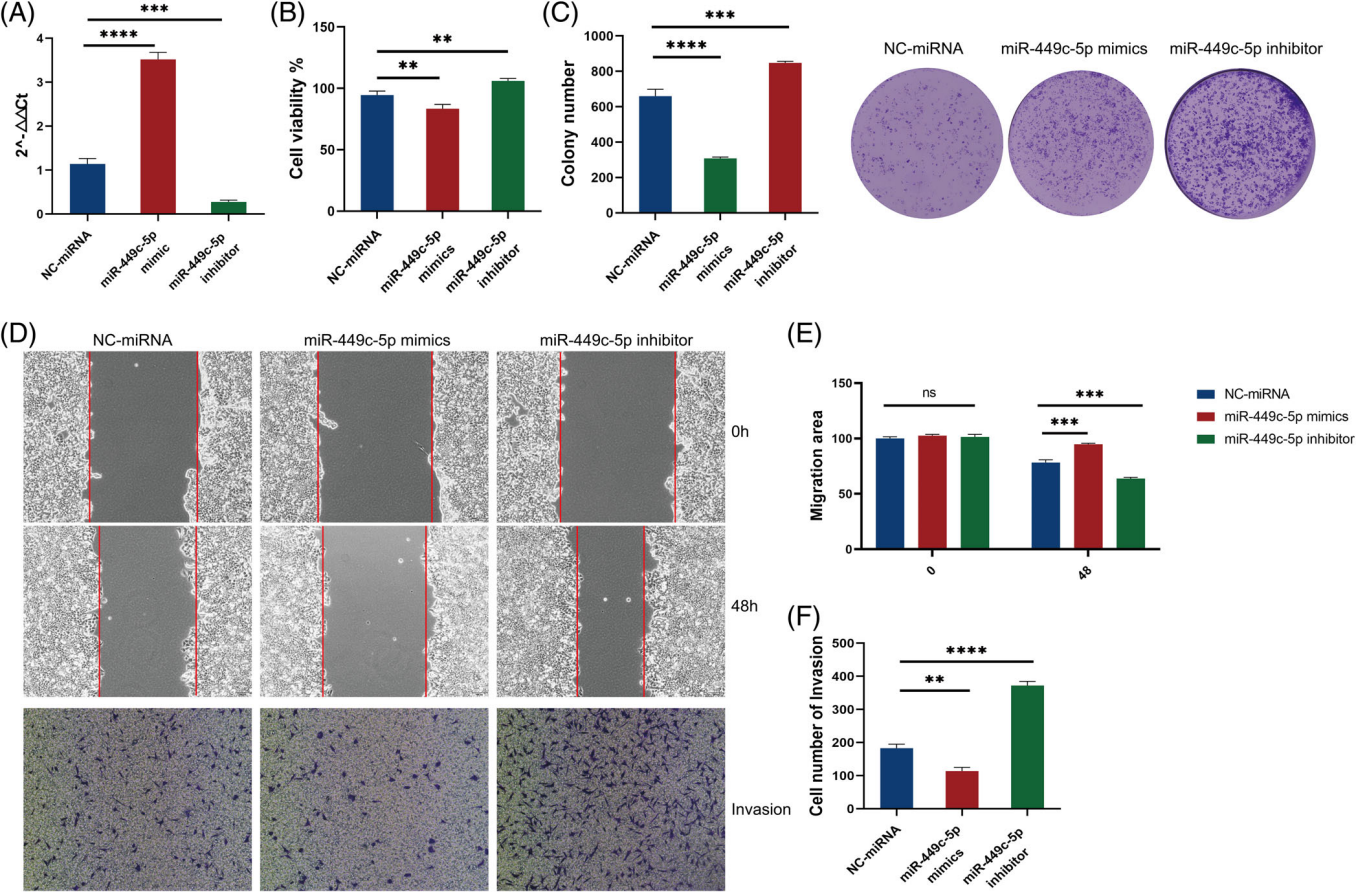
Breast cancer cell proliferation, migration and invasion ability influenced by miR‐449c‐5p. (A) Relative expression level in three groups by qRT‐PCR. (B) Colony formation to detect the colony cells. (C) Cell proliferation was measured by CCK‐8. (D) The migration area statistics results for the wound healing assay. (E) The Transwell assay's statistical results on invasion cell count. (F) Wound healing and Transwell were used to evaluate the cell migration and invasion. All data are shown as mean ± SD. n.s, not significant, ****P < .0001, ***P < .001, **P < .01, and *P < .05, one‐way ANOVA and two‐way ANOVA.

A series of experiments evaluated the role of miR‐449p‐5p in breast cancer progression. First, we transfected T47D cells with NC‐miRNA, miR‐449c‐5p mimics and, miR‐449c‐5p inhibitor, then detected the expression levels in three groups by qRT‐PCR (Figure 1A). It was found that the miR‐449c‐5p was higher expressed when miR‐449c‐5p mimics were transfected and lower expressed when the miR‐449c‐5p inhibitor was transfected which means the successful transfection of plasmids. The colony formation assay revealed that the overexpression of miR‐449c‐5p decreased the number of cell colonies, whereas the downregulation of miR‐449c‐5p resulted in an increase in colony formation (Figure 2B). Consistently, the CCK‐8 revealed that cell proliferation was reduced in the miR‐449c‐5p mimics group, but the miR‐449c‐5p inhibitor group increased the cell proliferation ability (Figure 2C). In addition, we measured the migration and invasion ability of breast cancer cells by Wound healing and Transwell separately (Figure 2D–F). The results elucidated that the overexpression of miR‐449c‐5p inhibited cell migration and invasion, while miR‐449c‐5p downregulation promoted these abilities. These experiments demonstrated that the overexpression of miR‐449c‐5p suppressed the proliferation, migration and invasion of breast cancer cells.

### 3.3 miR‐449c‐5p targeted ERBB2 in the breast cancer cells

**FIGURE 3:**
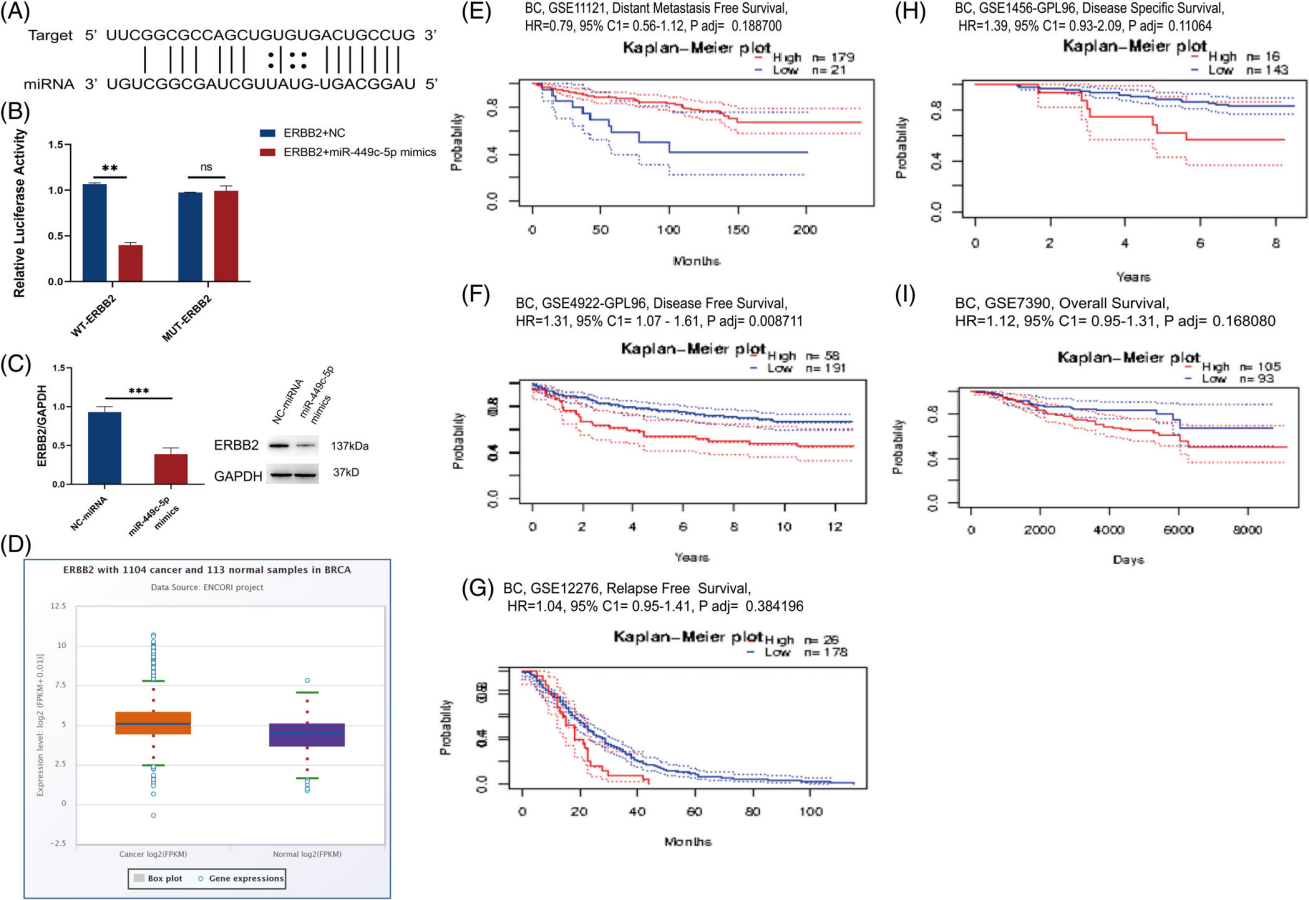
miR‐449c‐5p targeted ERBB2. (A) The predictive binding site of miR‐449c‐5p and ERBB2. (B) Luciferase activity of WT and MUT after co‐transfected with ERBB2 + miR‐449c‐5p into T47D cells. (C) Western blotting was used to investigate the expression level of ERBB2. (D) Relative expression of ERBB2 was evaluated in breast cancer tissues and normal tissues. (E) The distant metastasis survival curves with high or low ERBB2 expression. (F) The disease‐free survival curves with high or low ERBB2 expression. (G) The relapse survival curves with high or low ERBB2 expression.(H) The disease‐specific survival curves with high or low ERBB2 expression.(I) The overall survival curves with high or low ERBB2 expression. The results are presented as mean ± SD. n.s, not significant, ***P < .001, **P < .01 and *P < .05. Statistical significance was analysed by two‐way ANOVA and *t*‐test.

We then used Starbase to predict the potential target of miR‐449C‐5p to find the mechanism by which miR‐449c‐5p inhibits breast cancer progression (Figure 3A). A dual luciferase report assay was conducted to verify whether ERBB2 was target of miR‐449c‐5p. After the co‐transfection of miR‐449c‐5p mimics, the luciferase activity of cells in the WT‐ERBB2 group decreased. However, no changes were observed in the MUT‐ERBB2 group (Figure 3B). We also found the protein expression of ERBB2 in T47D cells was down‐regulated when the expression of the miR‐449c‐5p group increased (Figure 3C). Then, the gene expression of breast cancer from The Cancer Genome Atlas database (TCGA) showed an upward trend of the expression of ERBB2 in the cancer samples compared with normal samples (Figure 3D). These data indicated that ERBB2 is a target of miR‐449c‐5p, and miR‐449c‐5p negatively regulates the expression of ERBB2.

Moreover, we use Kaplan–Meier analysis and Log‐rank analysis to evaluate the traits of ERBB2 in terms of clinical prognosis. The distant metastasis survival was higher in the high expression of the ERBB2 group compared with the low expression group (Figure 3E). Also, it exhibited in other figures that the disease‐free survival, relapse‐free survival, disease‐specific survival and overall survival of the low expression group were higher than the high ERBB2 expression group (Figure 3F–I). Therefore, these data indicated that ERBB2 was a target of miR‐449c‐5p and a promoter of breast cancer.

### 3.4 The effect of miR‐449c‐5p/ERBB2 on proliferation, migration and invasion of breast cancer cells

**FIGURE 4:**
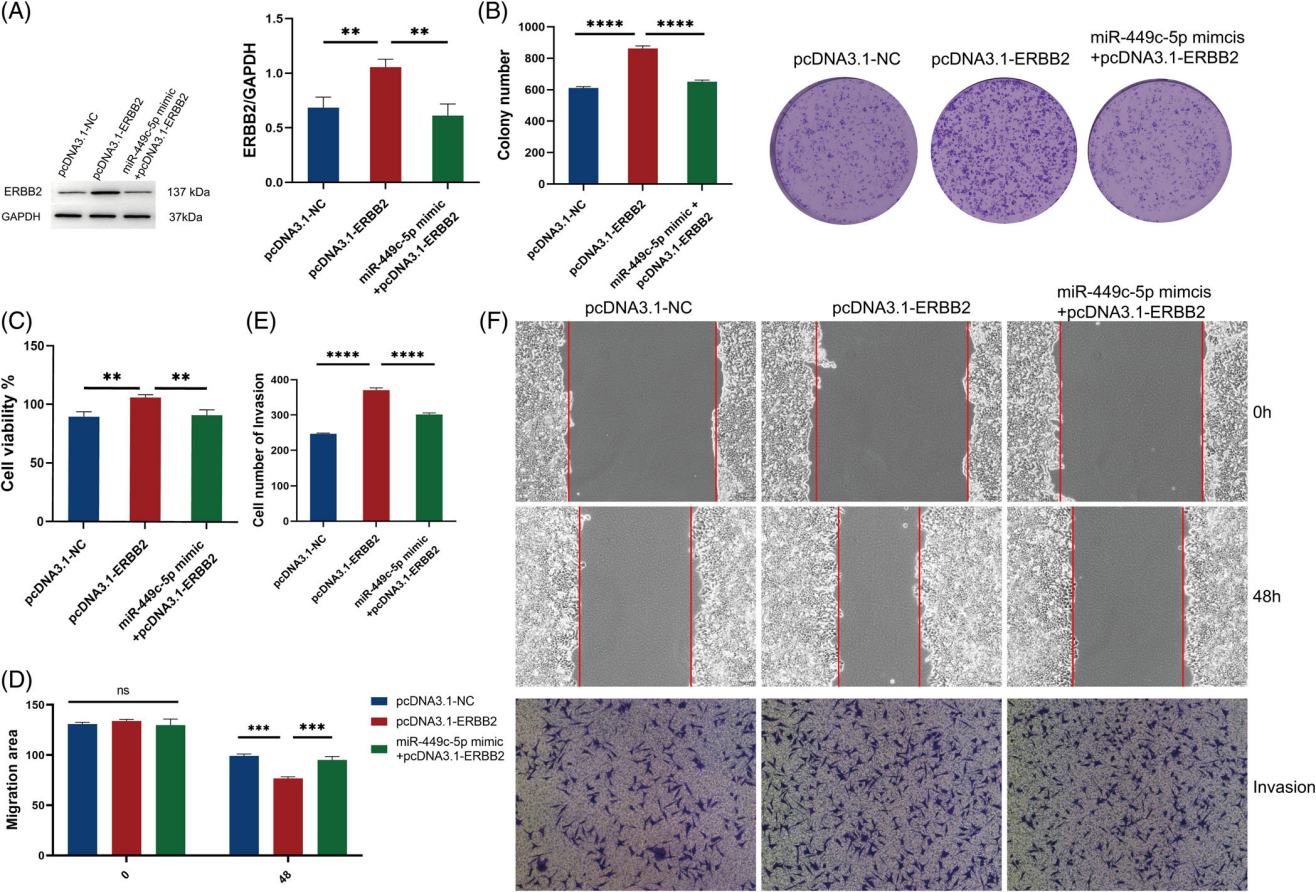
Breast cancer cell proliferation, migration and invasion ability affected by miR‐449c‐5p and ERBB2. (A) Expression level of ERBB2 in three groups by Western blotting. (B) Colony formation was measured to detect the colony cells. (C) Cell viability was measured by CCK‐8. (D) The statistical results of migration area for Wound healing assay. (E) The statistical results of invasion cell number for Transwell assay. (F) Cell migration and invasion detected by Wound healing and Transwell. The mean ± SD is provided. n.s, not significant, ****P < .0001, ***P < .001, **P < .01 and *P < .05, one‐way ANOVA and two‐way ANOVA.

To determine the effect of ERBB2 on breast cancer progression, we transfected T47D cells with pcDNA3.1‐NC, pcDNA3.1‐ERBB2, and miR‐449c‐5p mimic+pcDNA3.1‐ERBB2. The results of Western blotting showed protein expression of ERBB2 in T47D cells was reversed when the increased expression of the miR‐449c‐5p (Figure 4A). We also conducted Colony formation and observed that the overexpression of ERBB2 increased the colony number, whereas the miR‐449c‐5p+ERBB2 reversed this result (Figure 4B). CCK‐8 showed that ERBB2 overexpression increased cell proliferation in T47D cells, while these effects were restored by transfection with miR‐449c‐5p mimics (Figure 4C). Furthermore, Wound healing assay and Transwell assay were used to investigate cell migration and invasion ability (Figure 4D–F). The results showed that Cell migration and invasion were markedly increased in pcDNA3.1‐ERBB2, while the transfection of miR‐449c‐5p mimic reversed the promotion. These results demonstrated that miR‐449c‐5p regulated cell proliferation, migration and invasion through ERBB2.

### 3.5 miR‐449c‐5p regulating JAK‐STAT pathway by targeting ERBB2

**FIGURE 5:**
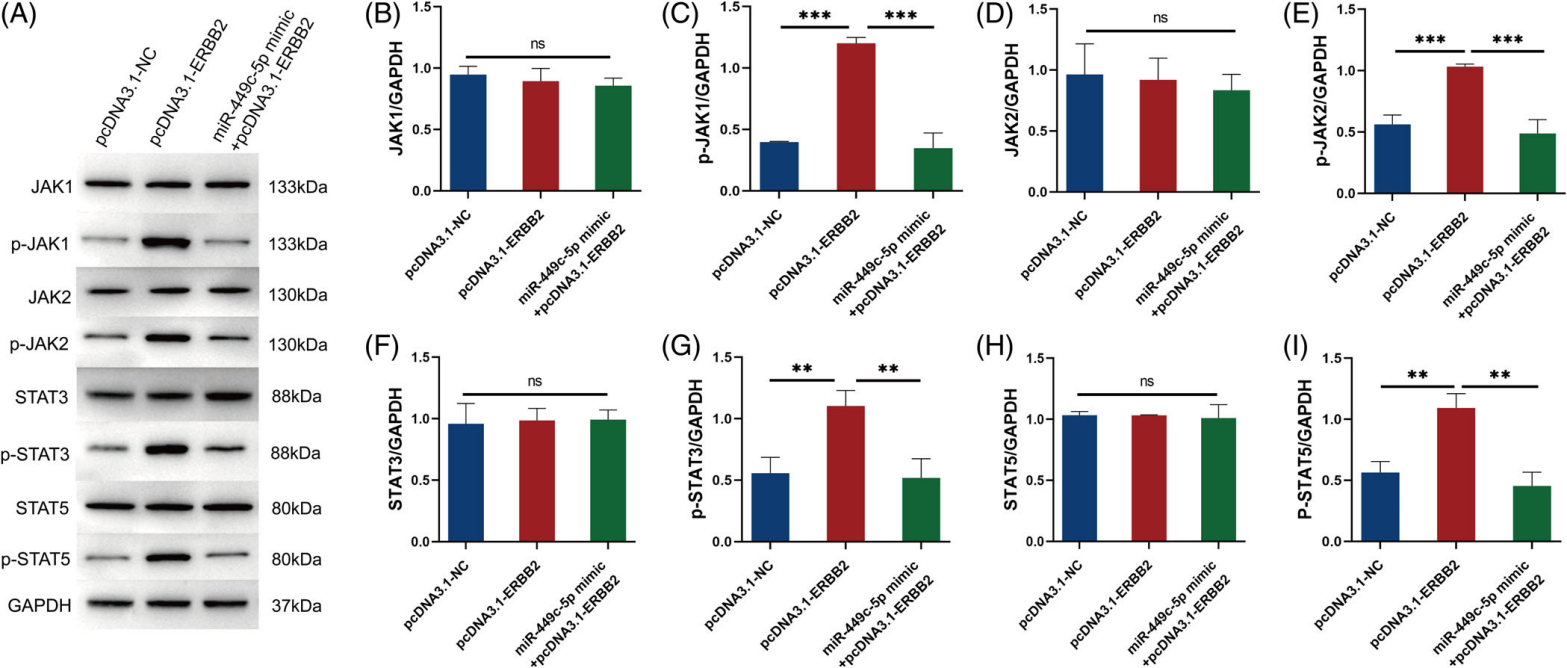
The protein expression of JAK1, p‐JAK1, JAK2, p‐JAK2, STAT3, p‐STAT3, STAT5, and p‐STAT5 was measured by western blotting in T47D cells after transfection with pcDNA3.1‐NC, pcDNA3.1‐ERBB2, and miR‐449c‐5p mimic+pcDNA3.1‐ERBB2. (A) The protein expression level of JAK1, p‐JAK1, JAK2, p‐JAK2, STAT3, p‐STAT3, STAT5, and p‐STAT5 by western blotting assay. (B–I) The statistical results of western blotting assay. Results are presented as the mean ± SD. n.s, not significant, ***P < .001, **P < .01 and *P < .05, according to one‐way ANOVA.

To confirm whether miR‐449c‐5p regulates the JAK‐STAT pathway, we used Western blotting to analyse the expressions of the JAK‐STAT pathway‐related proteins (JAK1, p‐JAK1, JAK2, p‐JAK2, STAT3, p‐STAT3, STAT5, and p‐STAT5). Here, we found that no evident fluctuations in JAK/STAT signalling pathway from Western blotting. Then, we found that ERBB2 resulted in an evident increase of p‐JAK1, p‐JAK2, p‐STAT3 and p‐STAT5 expression compared with pcDNA3.1‐NC, but the transfection of miR‐449c‐5p mimics restored the expression (Figure 5A–I); this means that miR‐449c‐5p targeted ERBB2 to regulate the JAK‐STAT pathway in the breast cell line.

## 4 DISCUSSION

Breast cancer is the highest incidence of malignancy worldwide; research^15^ in 2020 illustrated that breast cancer leads to 17% mortality. MiRNA binds to target mRNA in Untranslated Regions (UTR) and coding sequences (CDS), thus contributing too many biological processes. For example, in breast cancer, miR‐21 was extremely up‐regulated in both blood and tissues.^16^ In addition, it was shown in the research that miR‐30 was negatively correlated with prognosis.^17^ Reviews^18^, ^19^ indicated that miRNA modulates almost all stages of breast cancer progression. Thus, it is imperative to identify more miRNAs linked with breast cancer as novel biomarkers. Through investigation from the bioinformatics database (Starbase, PrognoScan), we found that miR‐449c‐5p overexpressed in breast cancer tissue compared to normal samples. The abnormal expression of miR‐449c‐5p was linked with a poor prognosis. Then, in our series of cell experiments, we found that miR‐449c‐5p was up‐regulated in breast cancer cells. We further investigated that overexpression of miR‐449c‐5p suppressed cell proliferation, colony formation ability, invasion, and migration. Therefore, miR‐449c‐5p has potential to be a novel biomarker for breast cancer.

Recent research found that miR‐449c‐5p regulated the proliferation and apoptosis of Glioblastoma (GBM).^20^ MiR‐449c‐5p was remarkably increased in palatal cells.^21^ It was a potential therapeutical target for chronic obstructive pulmonary disease (COPD), which was proved by a pilot study.^22^ In nasopharyngeal carcinoma (NPC), miR‐449c‐5p was differently expressed.^23^ The research articles indicated that miR‐449c‐5p regulated the STAT6 signalling pathway of acute cerebral infarction (ACI).^24^ However, the mechanism of miR‐449c‐5p in breast cancer lacks several pertinent research. In this research, we used the Starbase website and found a target of miR‐449c‐5p, ERBB2. The ERBB family is one type of receptor tyrosine kinases; it includes Human epidermal growth factor receptor 2 (ERBB2). ERBB2 (also known as HER2) leads to malignancies because of overexpression or amplified, particularly in breast cancer, bladder cancer, lung cancer, ovarian cancer, and so forth.^25^ It is overexpressed in about 25% of breast cancer. It relates to poor prognosis and chemo‐resistance.^26^ ERBB2 is an ideal target for breast cancer treatment. Overexpression of ERBB2 is found in primary tumors and the metastatic stage.^27^ It is a crucial receptor for breast cancer cell proliferation and a primary therapeutic target in cancer. Therefore, it is urgent to explore efficient clinical therapies targeted at ERBB2. In our research, ERBB2 was negatively correlated with miR‐449c‐5p, and its overexpression is associated with a poor prognosis. Furthermore, we found that miR‐449c‐5p/ERBB2 inhibited the proliferation, invasion and migration of breast cancer cells.

To further, investigate the role of miR‐449c‐5p in breast cancer. We learned that JAK/STAT is a communication node in the cell function, first discovered in 1990.^28^ This signalling pathway participated in cell proliferation, organ development and immune homeostasis.^29^ JAK/STAT consists of two ligand‐receptor complexes, which are JAKs and STATs. The JAK family includes JAK1, JAK2, JAK3, and TYK2. The STAT family composed of STAT1, STAT2, STAT3, STAT4, STAT5a, STAT5b, STAT6. Loss or mutation of JAK/STAT is related to many human diseases, such as malignancies and autoimmune diseases. A prospective clinical study^30^ also proved that JAK/STAT was a core regulator of breast cancer, and there is potential for combing chemotherapy drugs with this signalling pathway. Previous studies indicated that activation of the JAK/STAT pathway was dependent on HER2/HER3 heterodimerization.^31^ Thus, it is meaningful to find the upstream regulators of JAK/STAT to treat breast cancer. Here in our research, ERBB2 significantly increased JAK/STAT protein levels. In contrast, after being transfected with miR‐449c‐5p mimics, the protein level of JAK/STAT was restored. Taken together, miR‐449c‐5p targets ERBB2 through JAK/STAT signalling pathway.

In conclusion, our results show that miR‐449c‐5p suppresses breast cancer cell proliferation, invasion and migration by targeting ERBB2 via JAK/STAT signalling pathway. These findings may deliver new insight into breast cancer biomarkers. And the miR‐449c‐5p/JAK‐STAT/ERBB2 pathway may be a prospective therapeutic strategy for breast cancer. However, more in vivo animal experiments and clinical investigations are needed for miR‐449c‐5p in breast cancer. Recently, molecular studies, especially miRNA profiling, were used to improve breast cancer treatment and to assess prognosis, then found pivotal therapies.^32^ Although these technologies are still evolving, it is obvious that they have the potential to have an impact on breast cancer beyond the traditional clinical factors.^33^ Regarding chemical treatment, miR‐125b increased the drug sensitivity of 5‐FU.^34^ miR‐210 is associated with trastuzumab resistance in HER‐2+ breast cancer patients.^35^ Therefore, in the future, research might be focused on combining miR‐449c‐5p with chemotherapy.

## AUTHOR CONTRIBUTIONS

**Li Li:** Conceptualization (lead); writing – original draft (equal); writing – review and editing (equal). **Yangqiurong Zhang:** Data curation (equal). **Kunxian Yang:** Formal analysis (equal). **Wei Liu:** Writing – original draft (equal). **Ziting Zhou:** Writing – review and editing (equal). **Ying Xu:** Writing – review and editing (equal).

## FUNDING INFORMATION

This work was supported by Kunming Medical University Joint Specialized Fund 202101AY070001‐239.

## CONFLICT OF INTEREST STATEMENT

No benefits in any form have been or will be received from a commercial party related directly or indirectly to the subject of this manuscript.

## ETHICS STATEMENT

Not applicable.

## CONSENT FOR PUBLICATION

Some or all data, models, or codes generated or used during the study are available from the corresponding author by request.

## Data Availability

All data presented in this study were included in the article and supplementary files. Data is applicable after the approval of co‐authors.
